# Covalent Triazine Frameworks Decorated with Pyridine-Type Carbonitride Moieties: Enhanced Photocatalytic Hydrogen Evolution by Improved Charge Separation

**DOI:** 10.3390/polym15071781

**Published:** 2023-04-03

**Authors:** Xianxian Kong, Fan Yang, Xiaoying Li, Mengying Fu, Tao Zeng, Shuang Song, Zhiqiao He, Yan Yu

**Affiliations:** 1 College of Environmental, Zhejiang University of Technology, Hangzhou 310032, China; 2 College of Science & Technology, Ningbo University, Ningbo 315212, China

**Keywords:** pyridine-type carbonitrides, covalent triazine frameworks, photocatalytic hydrogen evolution reaction, separation efficiency

## Abstract

A simple procedure of calcination under an Ar atmosphere has been successfully applied to create a covalent triazine framework bearing pyridine-type carbonitride moieties (PCN@CTF). The appending of PCN on the CTF led to visible light absorption at up to 600 nm in the UV/Vis diffuse-reflectance spectra. Photoluminescence and electrochemical impedance spectroscopy have been applied to clarify how modification of the CTF with PCN enhanced the separation efficiency of photoexcited charge carriers. An optimized 1%PCN@CTF sample showed the highest photocatalytic hydrogen evolution reaction (HER) rate of 170.2 ± 2.3 μmol g^−1^·h^−1^, 3.9 times faster than that over the pristine CTF. The apparent quantum efficiency of the HER peaked at (7.57 ± 0.10)% at 490 nm. This representative 1% PCN@CTF sample maintained continuous function for at least 15 h. This work provides new guidance for modification with PCN materials as a means of obtaining high photocatalytic efficiency and sheds light on the effect of appended pyridine rings on a CTF.

## 1. Introduction

Hydrogen evolution from water by photocatalytic solar light-initiated photosynthesis has the potential to become one of the most alluring approaches toward meeting rising energy needs and addressing critical environmental problems [1,2,3]. In the past decades, numerous studies have been focused on exploring various types of inorganic photocatalysts, such as metal oxides [4], metal sulfides [5], and metal phosphides [6]. However, most of these photocatalysts have limited ultraviolet (UV) light response or exhibit moderate activity due to a relatively wide band gap and rapid electron–hole recombination [7,8]. Currently, organic and polymeric photocatalysts are attracting broad interest due to their tunable electronic structures and comparatively low cost [9,10,11].

Among the organic photocatalysts that have been reported to date, covalent triazine frameworks (CTFs) have shown the most promise for producing hydrogen gas (H_2_) when exposed to visible light [12,13,14]. Similar to other conjugated polymers, including two-dimensional graphitic carbon nitride (g-C_3_N_4_) and poly (triazine imide), CTFs have favorable properties, such as appropriate conduction band positions and easily tunable electronic structures [15]. Due to their highly porous, covalent, and conjugated nitrogen-rich structures, CTFs also have vast accessible surface areas and exceptional stability. It is significant that their aromatic rings are conjugated, which increases their capacity to absorb visible light, and that their stacked structures provide specific pathways for the transfer of photogenerated carriers [11,16]. The photocatalytic hydrogen evolution efficiency of CTFs is nevertheless poor due to the relatively rapid recombination rate of photogenerated charge carriers [17,18]. In this regard, a great deal of research effort has been devoted to retarding electron-hole recombination by means of element doping, dye-sensitization, and coupling with semiconductors.

Decorating semiconductor photocatalysts with carbonitrides, especially pyridine-type carbonitrides (PCN), has recently been proven to be an effective strategy for promoting photocatalytic hydrogen evolution activity [19,20]. For example, PCN/CTiO_2_@TiO_2−*x*_ and PCN-based donor-π-acceptor (D-π-A) networks have been shown to offer excellent hydrogen production performance [2,10]. Therefore, modification of CTFs with PCN appears to be an effective strategy for achieving satisfactory photocatalytic hydrogen production activity. Furthermore, to the best of our knowledge, research on such modification of CTFs has not been reported previously.

Based on the current state of the art, we report herein the successful synthesis of a novel PCN-decorated CTF (PCN@CTF) for photocatalytic water splitting under visible-light irradiation. The 4-aminopyridine (4-AP) was selected as a carbonization precursor in our experiments, which was expected to favor rapid electron mobility and prolong the lifetime of charge carriers. The obtained PCN@CTF was studied as a catalyst for H_2_ evolution under simulated sunlight and sacrificial conditions, using platinum (Pt) as a co-catalyst. In addition, comparative experiments were conducted at different synthesis temperatures in order to optimize its performance. Our results revealed a promising potential of PCN@CTF for highly efficient photocatalytic hydrogen evolution.

## 2. Experimental

### 2.1. Materials

All chemicals were used exactly as received. The 1,4-dicyanobenzene (DCB) was obtained from Beijing Bailingwei Technology Co., Ltd. (Beijing, China). Triethanolamine (TEOA) and 4-aminopyridine (4-AP) were obtained from Shanghai McLean Biochemical Technology Co., Ltd. (Shanghai, China). Ethanol, trifluoromethanesulfonic acid (TFMS), *N,N*-dimethylformamide (DMF), and chloroplatinic acid hexahydrate (H_2_PtCl_6_·6H_2_O) were obtained from Shanghai Aladdin Biochemical Technology Co., Ltd. (Shanghai, China). Ultrapure water utilized in the experiments was obtained from a Milli-Q^®^ ultrapure water purification system (Milford, MA, USA).

### 2.2. Catalyst Preparation

The CTF synthesis route consisted of three main steps. First, DCB (4 mmol) was added to TFMS (2.5 mL) under nitrogen at 0 °C, and the mixture was agitated for 2 h. The final product was collected after heating for 20 min at 100 °C, centrifuged, repeated washing with a mixture of water, ethanol, and ammonia, and overnight drying at 60 °C under vacuum.

CTF (0.5 g), ethanol (2 mL), and 4-AP were placed in a crucible and dried at room temperature. The precursor was then carbonized by heating in a tube furnace for 2 h at the requisite temperature of 400 °C, attained at a heating rate of 10 °C min^−1^. Catalyst powder samples denoted as *x*%PCN@CTF (*x* = 0.5%, 1%, 5%, and 10%), where *x* represents the different mole fractions of 4-AP, were collected after cooling to room temperature.

### 2.3. Characterization

The catalysts were subjected to X-ray diffraction (XRD) powder analysis on an X-ray diffractometer (XPert Pro MPD; PANalytical B.V., Almelo, The Netherlands) employing a Cu-*K*_α_ radiation source operated at 45 kV and 40 mA. Transmission electron microscopy (TEM) was carried out with a Tecnai G2F30 S-Twin TEM instrument (FEI, The Netherlands). A Thermo Scientific ESCA-Lab-200i-XL spectrometer (Waltham, MA, USA) was employed for X-ray photoelectron spectroscopy (XPS) employing monochromated Al radiation (1486.6 eV), and XPS Peak 4.1 software was employed to analyze the C 1s and N 1s spectra. A Nicolet Thermo NEXUS 670 spectrometer was utilized to acquire Fourier-transform infrared (FTIR) spectra over the range of 4000–400 cm^−1^ with a resolution of 4 cm^−1^. An ultraviolet-visible (UV/Vis) spectrophotometer (UV-2550; Shimadzu, Japan) was used to record UV/Vis diffuse-reflectance spectra over the wavelength range of 200–800 nm. Excitation at 500 nm was used to analyze photoluminescence (PL) spectra with a Hitachi F-4600 fluorescence spectrophotometer (Hitachi, Ibaraki, Japan). Software from Edinburgh Instruments integrated into the instrument was adopted to record and analyze the data. Nitrogen adsorption–desorption at −196 °C and a NOVA-2000E surface area analyzer (Quantachrome Corp, Boynton Beach, FL, USA) were used to compute Brunauer-Emmett-Teller (BET) surface areas.

### 2.4. Photoelectrochemical and Electrochemical Measurements

All photoelectrochemical (PEC) and electrochemical (EC) measurements were carried out on a CHI 760E electrochemical workstation (CH Instruments, Austin, TX, USA) using a conventional three-electrode system, consisting of an Ag/AgCl reference electrode, a Pt plate (1 cm^2^) as the counter electrode, and fluoride-tin oxide (FTO) with a photocatalyst coating as the working electrode. To generate a homogeneous catalyst colloid for these experiments, sample (20 mg) and Nafion^®^ (50 μL) were agitated in isopropanol (300 μL) for at least 15 min. The catalyst colloid (90 μL) was then drop-cast onto clean, approximately 1 cm^2^ FTO conductive glass plates and allowed to dry in air. The lighting source and electrolyte were a 300 W xenon lamp from the Beijing Electric Light Sources Research Institute in China and a 0.1 mol L^−1^ Na_2_SO_4_ solution, respectively.

The photocurrent response in the on-off state was detected by acquiring *i*-*t* curves at a horizontal potential of 1.23 V vs. Reversible Hydrogen Electrode (RHE). Electrochemical impedance spectroscopy (EIS) was carried out in complete darkness utilizing an open-circuit potential with an alternating current voltage of magnitude 5 mV over the frequency range 10^−1^–10^6^ Hz. The arc in the impedance spectra was fitted using a condensed Randles equivalent circuit comprising a resistance (R_s_, electrical conductivity of the electrodes), a charge-transfer resistance (R_ct_, interface electrocatalytic reaction between the electrode and electrolyte), and a constant-phase element. The Levenberg-Marquardt minimization method was used to estimate the fitting parameters.

### 2.5. Photocatalytic Hydrogen Evolution

A powdered photocatalyst (50 mg) was dispersed in 100 mL of an aqueous solution containing 10 vol% TEOA and 2 vol% H_2_PtCl_6_ for each reaction. After being sonicated for 30 min, the catalyst suspension was subjected to solar radiation for 1 h in a solar simulator equipped with an AM 1.5G filter and a 300 W Xe lamp. This allowed for the photodeposition of 3 wt% Pt, which served as a co-catalyst, on the catalyst surface. The system was completely purged of air for 30 min prior to the photocatalytic H_2_ production experiment. The light source was then turned on, and the suspension was continuously stirred to ensure that the particles were evenly illuminated. The gaseous products were directed to a 7890B gas chromatograph (GC; Agilent, Santa Clara, CA, USA) at specified intervals to quantitatively calculate the amount of H_2_ generated. The GC was equipped with a thermal conductivity detector and an HP-Molesieve capillary column (30 m × 0.32 mm × 12 μm). The photocatalytic performance was plotted against incident light wavelength using 370, 400, 430, 460, 490, and 520 nm band-pass filters. The quantum efficiency (QE) for H_2_ evolution was then calculated according to Equation (1):

(1) $$\mathrm{QE}\;\left(\%\right)=\frac{{\mathrm{number}\;\mathrm{of}\;H}_{2}\;\mathrm{molecules}\;\mathrm{generated}\;\times 2}{\mathrm{number}\;\mathrm{of}\;\mathrm{incident}\;\mathrm{photons}}$$

## 3. Results and Discussion

### 3.1. TEM and XRD

**Figure 1:**
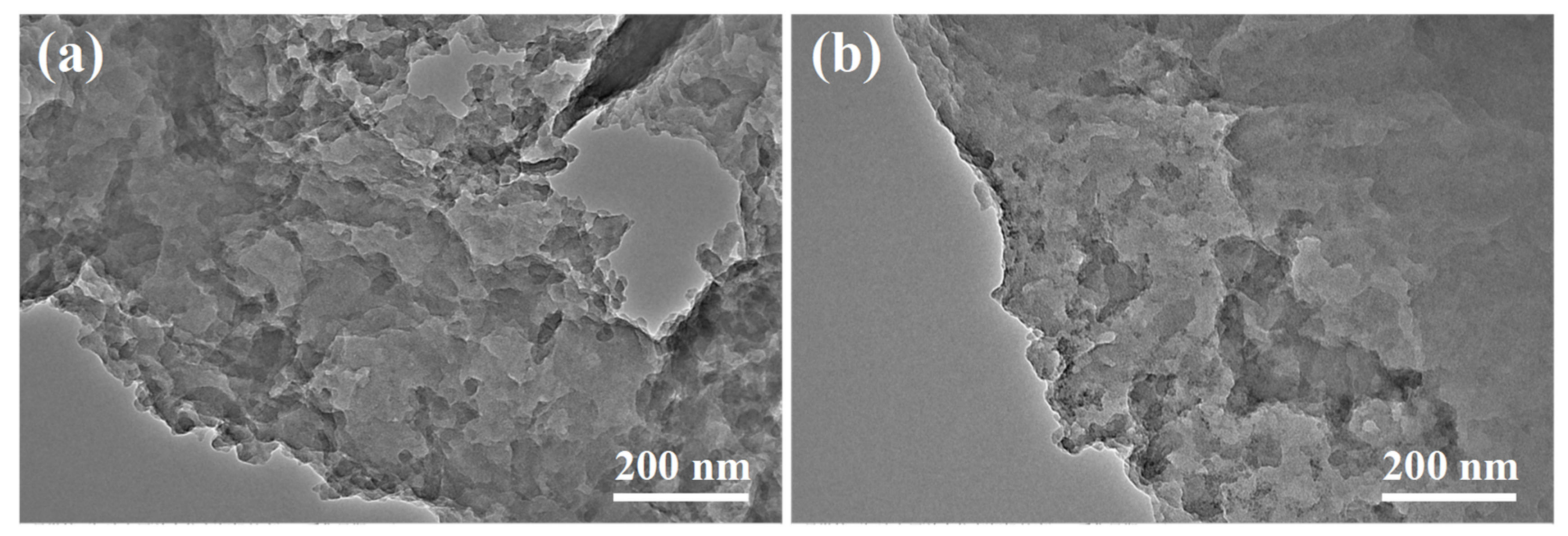
TEM images of (**a**) CTF and (**b**) 1%PCN@CTF.

**Figure 2:**
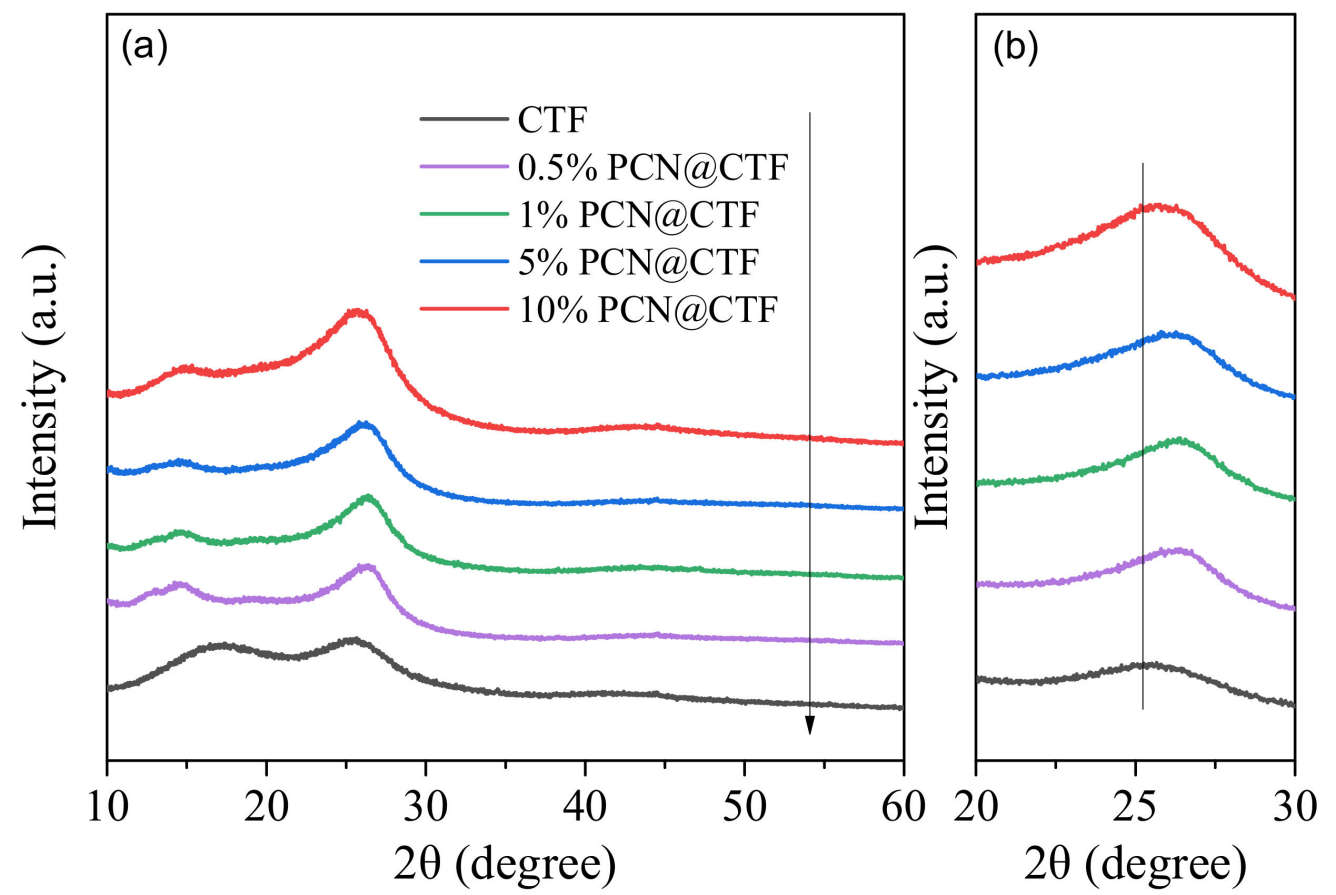
XRD patterns of (**a**) CTF and (**b**) 1%PCN@CTF.

Morphological and structural aspects of the samples were probed by TEM analysis. As illustrated in Figure 1, TEM images of both pristine CTF and 1%PCN@CTF showed an analogous typical nanosheet structure [21]. As shown in Figure 2a, the pure CTF sample displayed two XRD peaks at 15.9° and 25.7°, attributable to the (002) plane resulting from interlayer stacking of the π-conjugated rings and the (002) facet representing the (100) facet corresponding to the in-plane long-range molecular order of the polymer network, respectively [22,23]. The similar diffraction patterns of the *x*%PCN@CTF samples suggested that the appending of the pyridine rings had little effect on the crystalline structure of CTF. Enlarged XRD patterns of the *x*%PCN@CTF samples (Figure 2b) showed that the appending of PCN lowered the interlayer stacking distance, which caused the (002) diffraction peaks to shift to higher angles in comparison to the pristine CTF [23].

### 3.2. FTIR

**Figure 3:**
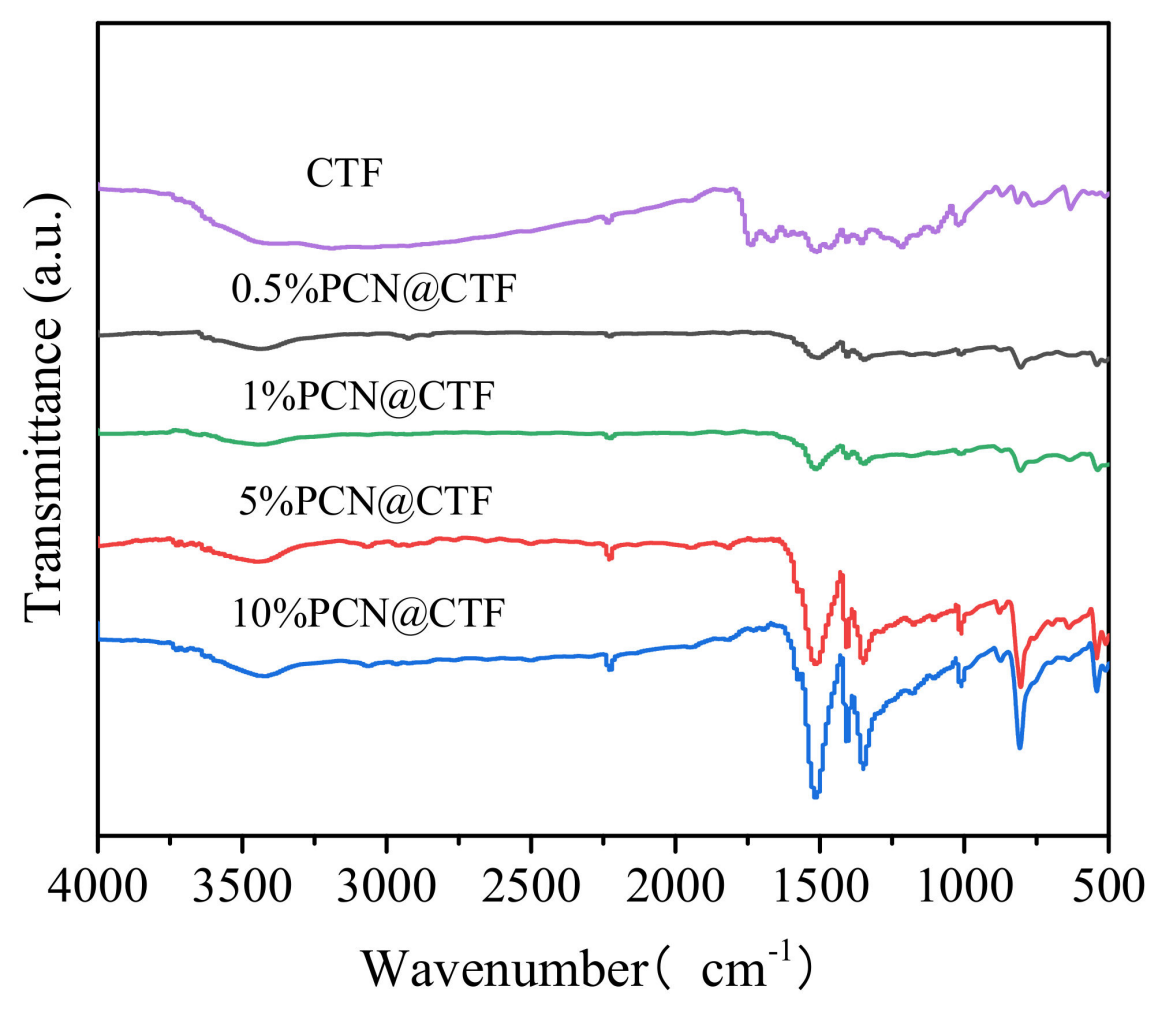
FTIR spectra of CTF and *x*%PCN@CTF.

FTIR spectra (Figure 3) were acquired to examine the chemical structures of the materials. Analysis revealed that C-H stretching vibrations gave rise to characteristic peaks at 810, 1011, 2859, and 2924 cm^–1^ [24]. Additionally, two prominent absorption bands at 1501 and 1345 cm^–1^ indicated the presence of triazine rings. Peaks at 1500–1480 and 1600–1590 cm^–1^ could be assigned to phenyl groups. The broad peak at 3300–3690 cm^–1^ could be assigned to the stretching vibrations of terminal amine groups (-NH_2_) [25]. Notably, these characteristic peaks were found to be almost identical, irrespective of the samples tested, implying that modification with PCN has minimal effect on the molecular structure of CTF.

### 3.3. UV/Vis Diffuse-Reflectance Spectroscopy

**Figure 4:**
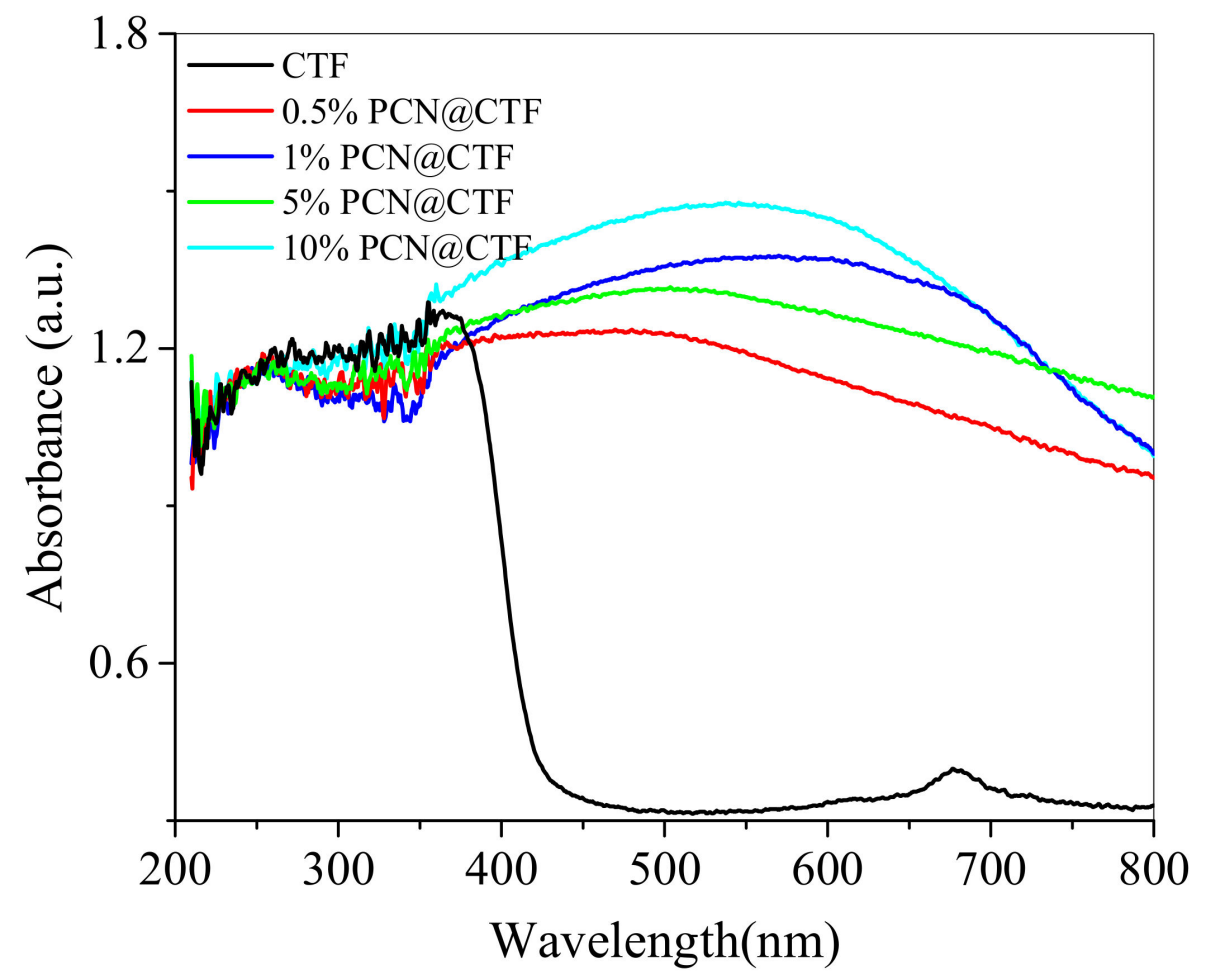
UV/Vis absorption spectra of CTF and *x*%PCN@CTF.

The ability of the photocatalysts to absorb light was confirmed by the UV/Vis diffuse-reflectance spectra presented in Figure 4. The *x*%PCN@CTF samples displayed improved absorption in the 400–550 nm range, whereas no discernible absorption was observed for pristine CTF. The appending of PCN on CTF gave rise to an absorption shoulder, indicating enhanced photon absorption. Notably, *x*%PCN@CTF exhibited significantly higher absorbance intensity across the visible-light spectrum compared to pure CTF, which could only be attributed to the protective PCN layer on the CTF surface.

### 3.4. BET Surface Area

**Figure 5:**
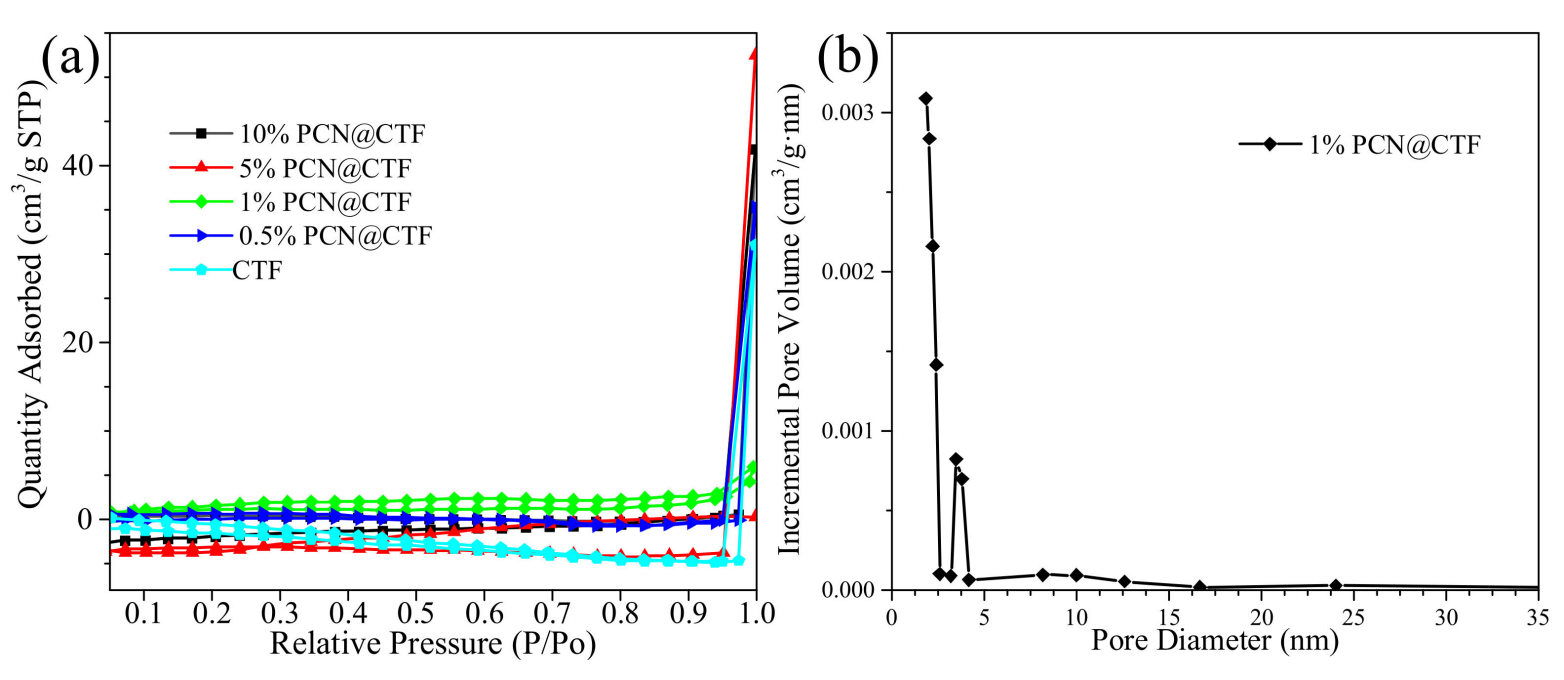
(**a**) N_2_ adsorption/desorption isotherms (77 K) and (**b**) desorption pore size distribution plots of CTF and *x*%PCN@CTF.

The precise surface areas and porosities of the prepared samples were determined by the N_2_ adsorption-desorption technique, as shown in Figure 5. The as-obtained samples displayed typical type IV isotherms and discrete hysteresis loops (H3-type) in a high relative pressure range (*p*/*p*_0_, 0.61–1.0), showing the presence of slit-like mesopores and capillary condensation [26]. The surface areas of pure CTF, 0.5%PCN@CTF, 1%PCN@CTF, 5%PCN@CTF, and 10%PCN@CTF were 0.10, 0.31, 3.26, 2.33, and 1.19 m^2^ g^−1^, respectively. Thus, compared with pristine CTF, the *x*%PCN@CTF samples attained increased BET surface areas, confirming that PCN had been successfully appended on the CTF and modified its porous structure and surface area. The 1%PCN@CTF sample displayed a relatively larger major pore (Figure 5b). With a sizeable amount of PCN adsorbed on the substrate, the hierarchical structure with a larger surface area apparently provided more catalytically active sites [25].

### 3.5. XPS

**Figure 6:**
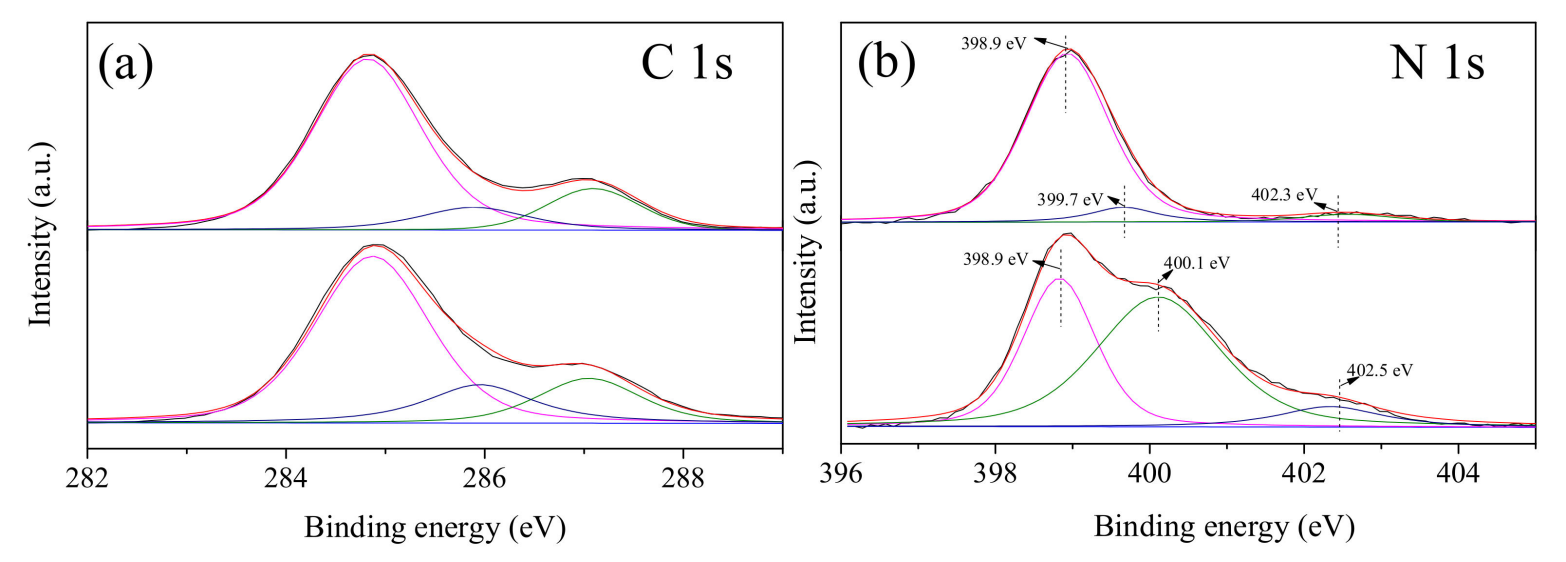
XPS patterns of CTF and 1%PCN@CTF: (**a**) C 1s and (**b**) N 1s.

Deconvolution of the C 1s peaks (Figure 6a) of CTF and 1%PCN@CTF revealed three primary peaks at 287.1, 285.9, and 284.6 eV. These peaks could be attributed to the presence of C=O, C=N, and π–π* bonds, respectively, as reported in previous studies [27,28]. The N 1s pattern of CTF (Figure 6b) showed three peaks centered at 398.9, 399.7, and 402.3 eV, attributable to sp^2^-hybridized nitrogen (N-C=N/C-N=C), tertiary nitrogen (N-C_3_), and terminal amino groups (C-NH_2_), respectively, in accordance with reported studies [25]. Remarkably, the peaks assigned to N-C_3_ and C-NH_2_ in the 1%PCN@CTF sample shifted to higher binding energies, indicating a reduction in the electron density around the nitrogen atoms due to the appending of electron-withdrawing pyridine rings on the CTF framework. Additionally, compared with CTF, the increased carbon content in 1%PCN@CTF samples suggested the significant changes in carbon structure induced by PCN.

### 3.6. Photoluminescence

**Figure 7:**
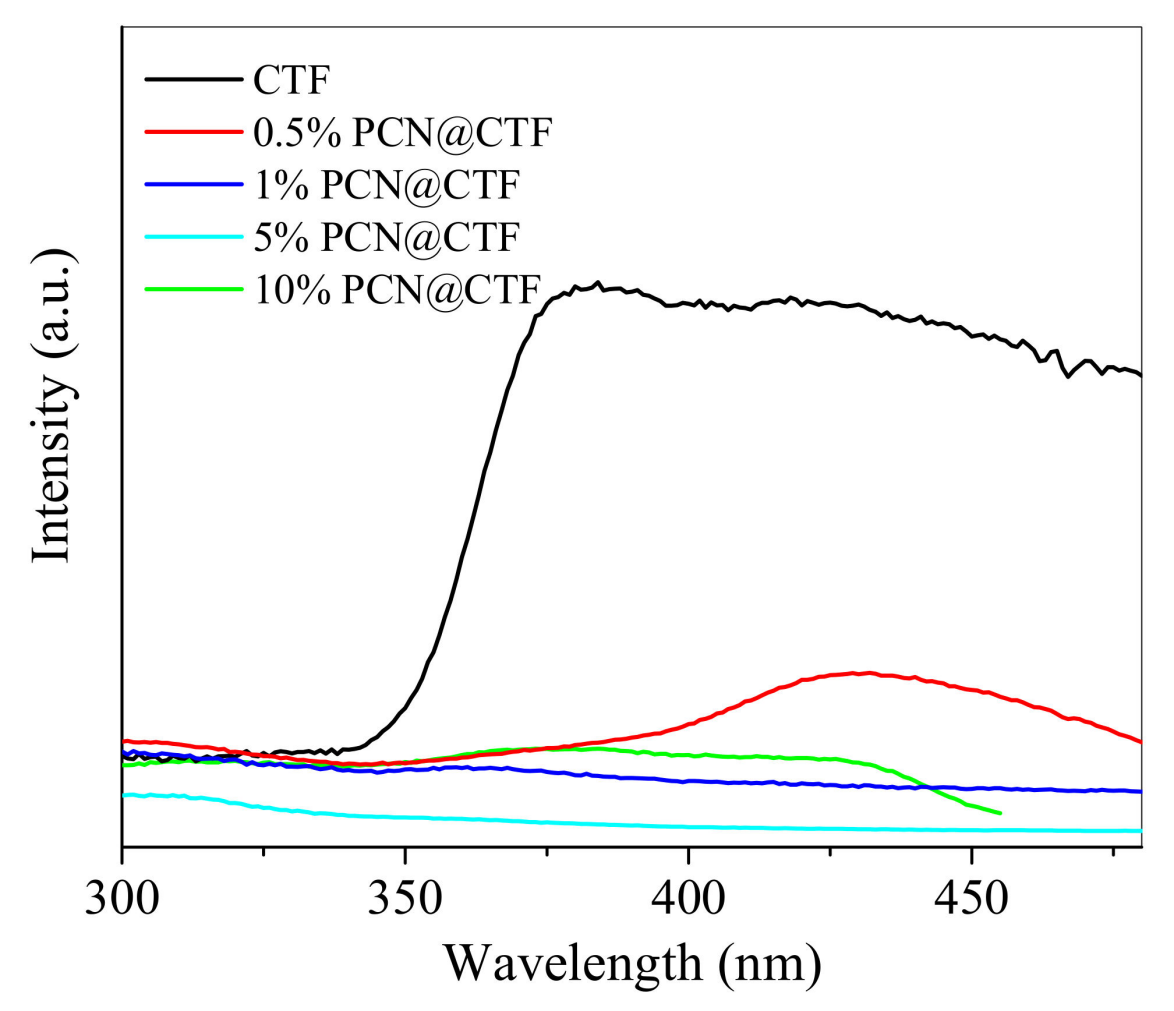
Stable photoluminescence spectra of CTF and *x*%PCN@CTF.

Figure 7 displays the fluorescence spectra of pure CTF and the *x*%PCN@CTF photocatalysts, each acquired at an excitation wavelength of 325 nm. Notably, the 5%PCN@CTF sample showed the lowest PL signal peak intensity among all of the samples, indicating maximum efficiency in separating photoinduced electron–hole pairs [29]. The 1% PCN@CTF sample also showed a weak PL signal. Appropriate preparation of the photocatalytic system enabled the successful promotion of photogenerated charge carrier separation, which effectively increased the photocatalytic activity. It is noteworthy that, compared to pure CTF, the *x*%PCN@CTF catalysts produced fluorescence peaks with substantially lower intensity.

### 3.7. Analysis of i-t Characteristics

**Figure 8:**
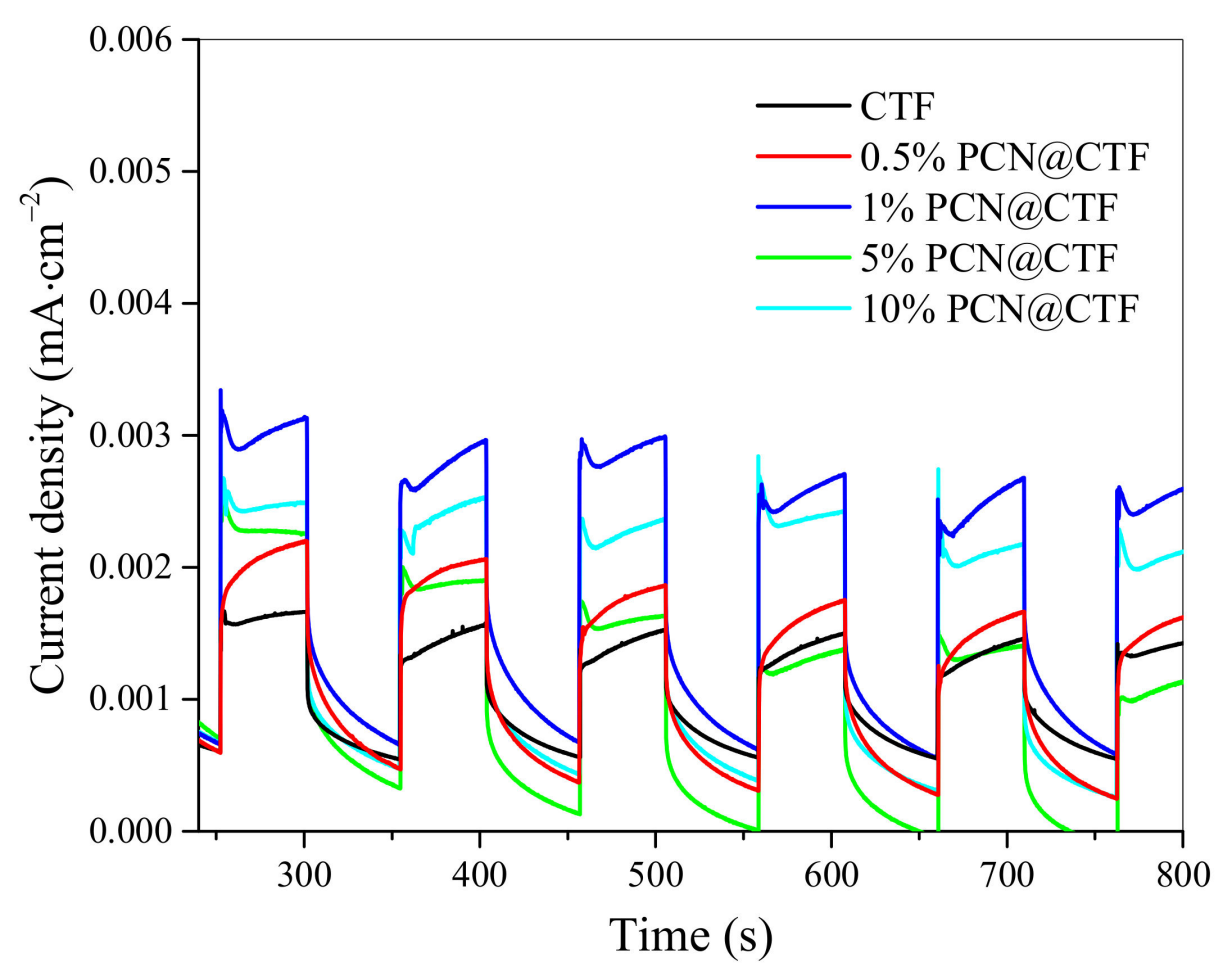
Periodic ON/OFF photocurrent responses of CTF and *x*%PCN@CTF.

The photocatalytic reaction, which is indirectly represented by photophysical, electrochemical, and photoelectrochemical properties, is caused by efficient and rapid transmission and separation of photoinduced charge carriers [30]. Figure 8 displays photocurrent plots of the CTF and *x*%PCN@CTF composites in their as-prepared states. As displayed in Figure 8, a photocurrent density of 1.5 μA cm^−2^ was recorded for the pristine CTF, reflecting a poor separation ability of electrons and holes. The highest photocurrent, 3.27 μA cm^−2^, was observed for the 1%PCN@CTF composite, about 2.2 times that for pristine CTF, showing that the separation and transfer of photogenerated charge carriers had been much improved. The photoelectrochemical properties and hydrogen evolution reaction (HER) activities are clearly linked, since the photocurrent response intensities closely match the photocatalytic HER rates.

### 3.8. EIS

**Figure 9:**
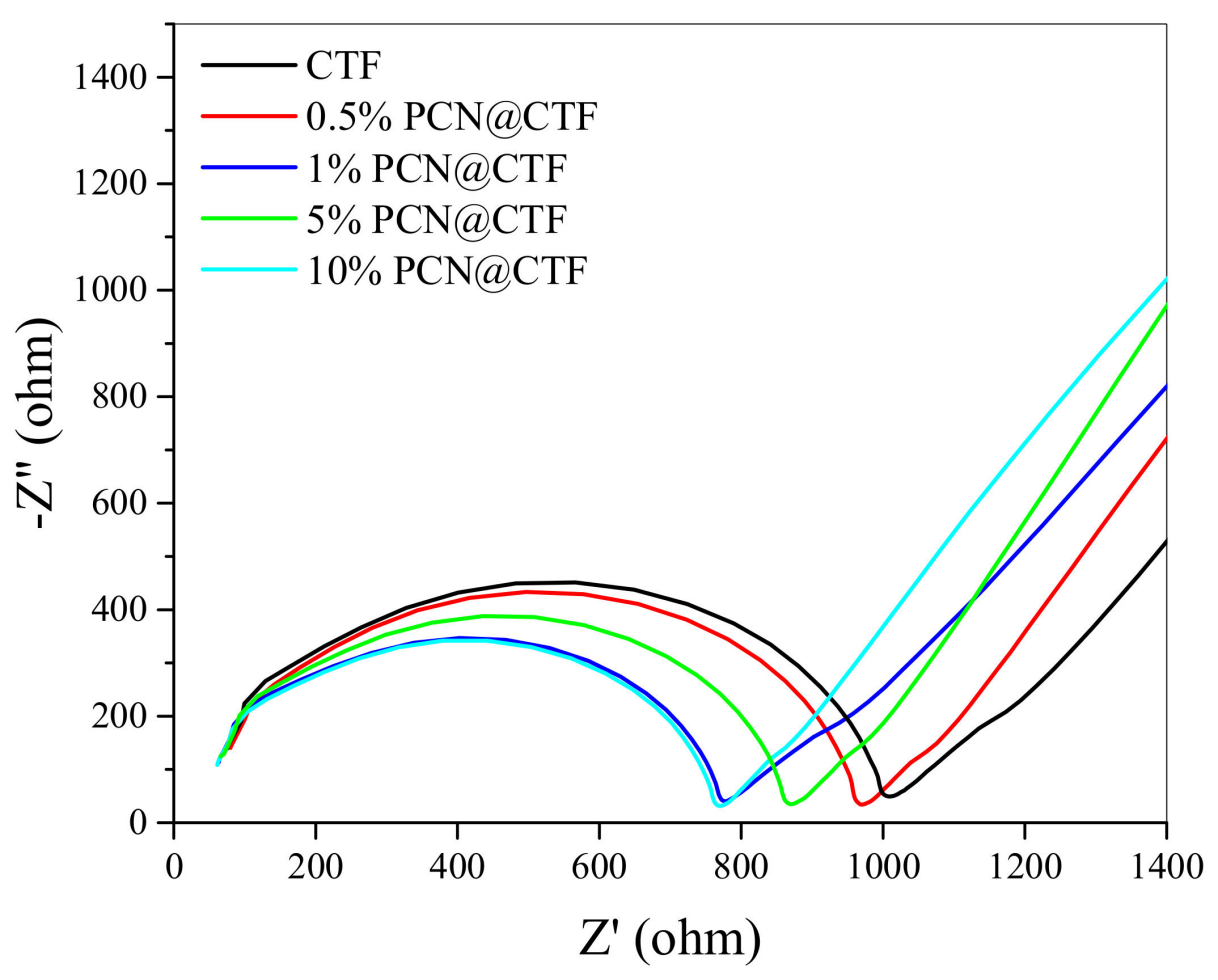
EIS Nyquist plots measured under an open-circuit potential of 5 mV for CTF and *x*%PCN@CTF.

Additionally, electrochemical impedance spectroscopy (EIS) was conducted in order to elucidate interfacial electron mobility [31]. As demonstrated in Figure 9, the EIS Nyquist plot featured a smaller semi-circle for the 1%PCN@CTF electrode, indicating a lower electrical resistance and a faster interfacial charge-transfer capability than those of the primary CTF electrode. Thus, the charge-transfer resistance on the 1%PCN@CTF surface was decreased, resulting in the effective separation of e^−^–h^+^ pairs, consistent with the findings from *i*-*t* analysis. During the photocatalytic reaction procedure, the 1%PCN@CTF photocatalyst showed the highest separation and migration efficiency, as demonstrated by the abovementioned data, far exceeding that of the pristine CTF.

In summary, the aforementioned photophysical, electrochemical, and photoelectrochemical properties suggested that the appending of PCN on the CTF facilitated the attainment of improved photocatalytic HER performance by enhancing the transfer and separation efficiency of charge carriers.

### 3.9. Photocatalytic Hydrogen Evolution

**Figure 10:**
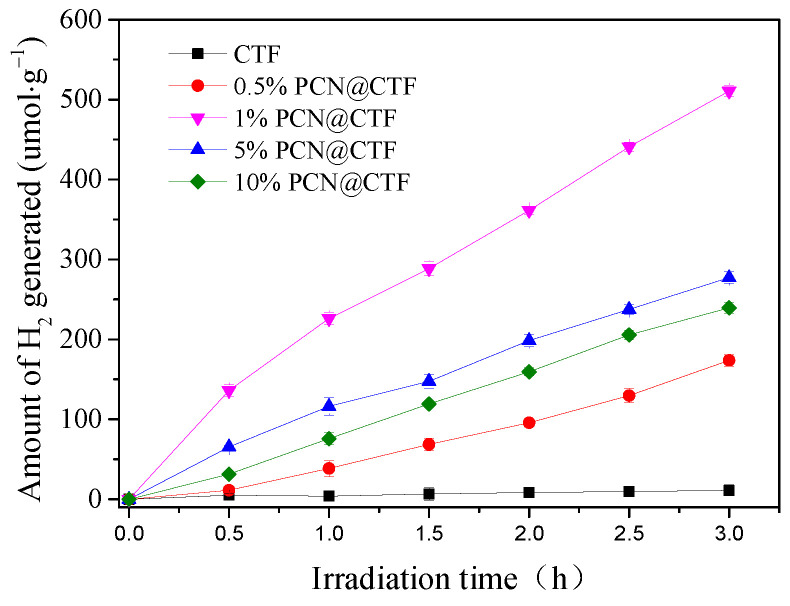
Photocatalytic hydrogen evolution reaction using CTF and *x*%PCN@CTF under light illumination.

The photocatalytic HER activities of the samples were assessed under visible-light illumination (λ > 420 nm). Figure 10 shows that the photocatalytic HER performances of the *x*%PCN@CTF samples increased dramatically with appropriate amounts of 4-AP. Specifically, the 1%PCN@CTF sample had the highest HER rate of 170.2 ± 2.3 μmol g^−1^ h^−1^, almost 46 times higher than that of the pristine CTF (3.7 ± 2 μmol g^−1^ h^−1^). The activities of the *x*%PCN@CTF catalysts decreased in the order 1%PCN@CTF > 5%PCN@CTF > 10%PCN@CTF > 0.5%PCN@CTF. Clearly, the decoration of PCN on the CTF was beneficial for enhancing the electronic excitation effect of visible light. As is displayed in Table 1, this value was comparable or superior to similar graphite-like carbon nitride catalysts previously reported in relation to the visible-light water-splitting process.

**Figure 11:**
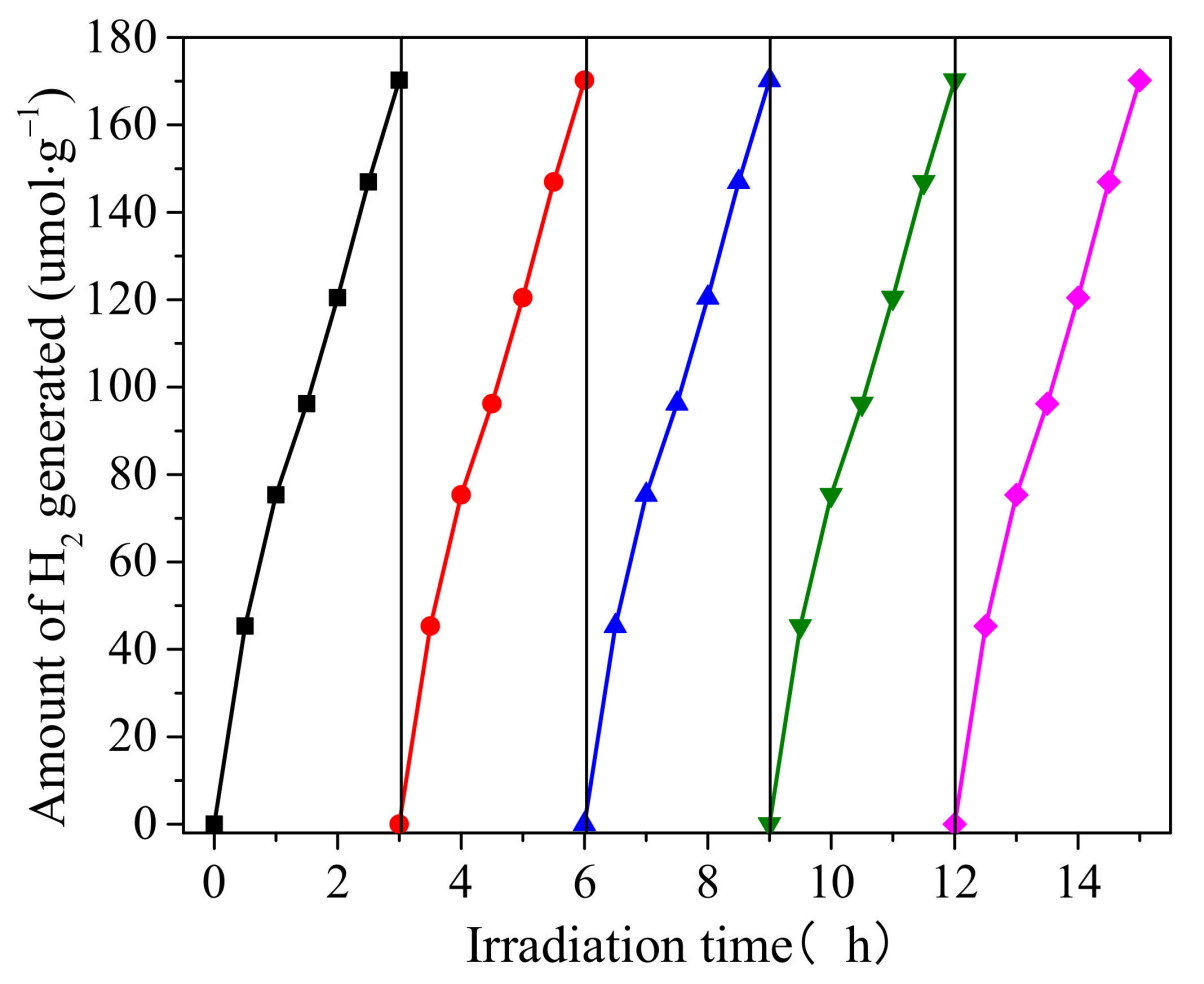
The stability of 1%PCN@CTF under light irradiation.

To understand the relationship between photoactivity and light absorption of 1%PCN@CTF, we examined the quantum efficiency (QE) for the evolution of H_2_ from water as a function of the wavelength of the incident light. The calculated QE values were (5.91 ± 0.10)%, (6.90 ± 0.15)%, (6.70 ± 0.12)%, (7.08 ± 0.13)%, (7.57 ± 0.10)%, and (7.23 ± 0.12)% at wavelengths of 370, 400, 430, 460, 490, and 520 nm, respectively. Figure 11 illustrates the correlation between QE and the UV/Vis absorption spectrum of 1%PCN@CTF. The wavelength dependence of QE was consistent with photo-absorption by the catalyst initiating the reaction.

**Figure 12:**
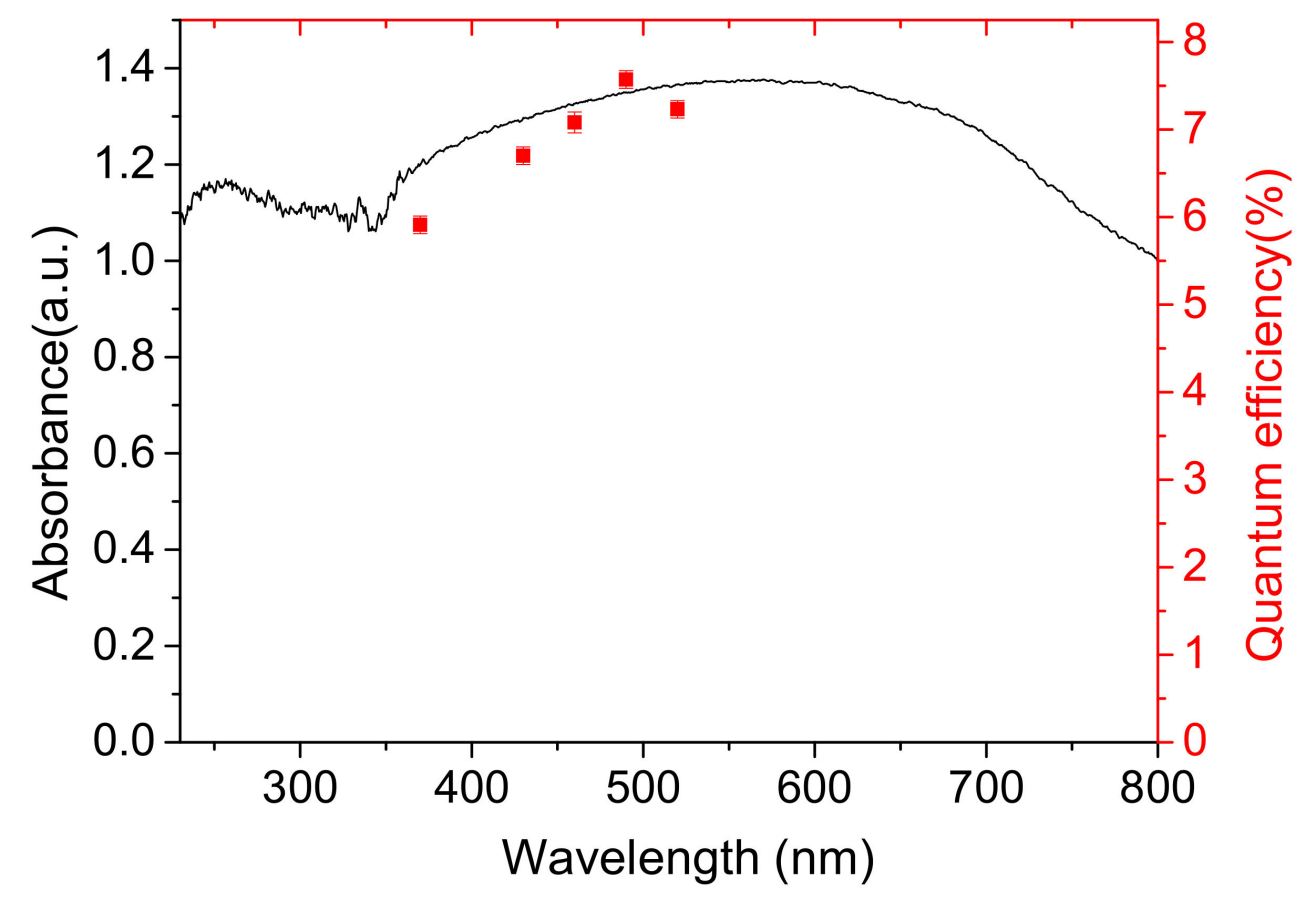
Wavelength-dependent QE for H_2_ evolution using 1%PCN@CTF and the UV/Vis absorption spectrum of 1%PCN@CTF.

The photocatalytic stability of a catalyst is clearly essential for its long-term utility. The 1%PCN@CTF sample was thus subjected to recycling procedures. The test was run cumulatively under visible-light irradiation for 15 h, with evacuations after every 3 h. It was not necessary to replenish the initial amount of methanol because it was sufficient for the duration of the entire activity test. The average linear H_2_ evolution rate measured over 1%PCN@CTF for 15 h was 168.9 ± 2.1 μmol h^−1^ g^−1^, with a standard deviation of 3.3%, as shown in Figure 12. Thus, the 1%PCN@CTF photocatalyst demonstrated continuous and sustained H_2_ evolution without any loss of photocatalytic activity.

**Scheme 1:**
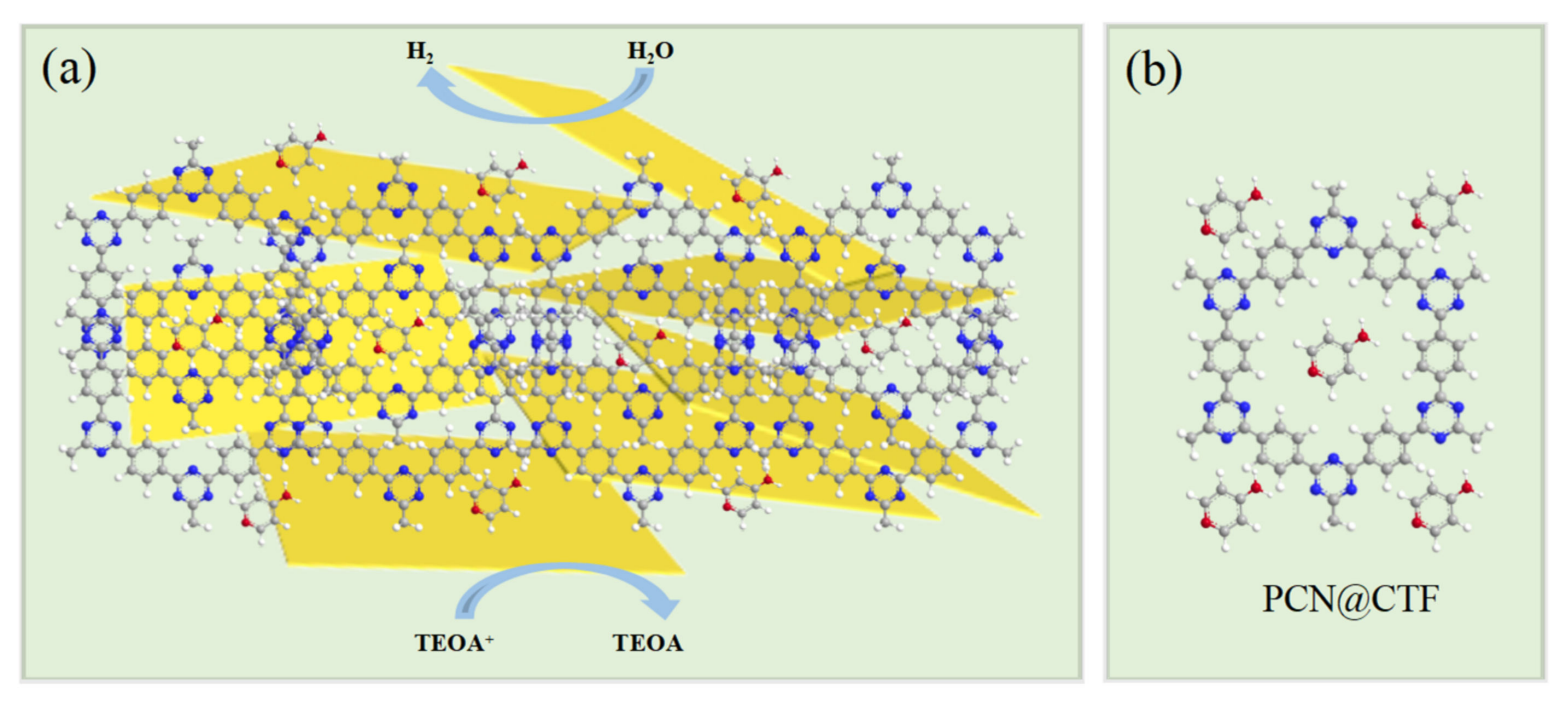
(**a**) Schematic illustration of the photocatalysis mechanism for hydrogen evolution; (**b**) the structure of PCN@CTF.

A possible photocatalytic mechanism for the HER over the 1%PCN@CTF sample is proposed. As depicted in Scheme 1, the electron-withdrawing pyridine rings facilitate the transfer of photogenerated electrons from the CTF to the PCN, thereby enhancing the separation efficiency of electron-hole pairs. Upon introducing Pt nanoparticles onto the CTF, photogenerated electrons are immediately transferred and accumulate at the Pt sites, while photogenerated holes remain in the CTF. Subsequently, water molecules are reduced by the photogenerated electrons to generate hydrogen, while the photogenerated holes are selectively consumed by TEOA in the solution [25,37].

## 4. Conclusions

In conclusion, a one-step hydrothermal procedure has been successfully applied to produce novel *x*%PCN@CTF catalysts. Such materials served as very effective and reliable photocatalysts for producing hydrogen from water using solar energy. The 1%PCN@CTF catalyst showed the highest catalytic activity, giving an H_2_ evolution rate of 170.2 ± 2.3 μmol h^−1^ g^−1^ when exposed to light. At 490 nm, the QE for H_2_ generation on 1%PCN@CTF was (7.57 ± 0.10)%. Charge-separation efficiency may be improved by the appending of electron-withdrawing PCN on the CTF to boost photocatalytic H_2_ evolution.

## Figures and Tables

**Table 1:** A comparison of the hydrogen evolution performance of PCN@CTF with other graphite-like carbon nitride materials.

Photocatalyst/ Co-Catalyst	Light Source	Aqueous Reaction Solution	H_2_ Evolution Rate	Reference
PCN-9-Pt	>400 nm	TEOA	33.5 μmol g^−1^ h^−1^	Ref. [32]
COF–imide	>420 nm	Water	34 μmol h^−1^∙g^−1^	Ref. [33]
N_1_–COF	>420 nm	PBS buffer	90 μmol h^−1^∙g^−1^	Ref. [34]
N_0_–COF	>420 nm	PBS buffer	23 μmol g^−1^ h^−1^	Ref. [34]
Cu_2_O@C_3_N	Visible light	EDTA	26 μmol g^−1^ h^−1^	Ref. [35]
g–C_33_N_3_–COF	>420 nm	Water	74 μmol h^−1^∙g^−1^	Ref. [36]
CTF	AM 1.5G	TEOA	3.7 ± 2 μmol g^−1^ h^−1^	This work
1%PCN@CTF	AM 1.5G	TEOA	170.2 ± 2.3 μmol g^−1^ h^−1^	This work

## Data Availability

The data that support the findings of this study are available on request from the corresponding author, Yan YU, upon reasonable request.
